# The Analysis of Physiological Variations in M_2_ Generation of *Solanum melongena* L. Mutagenized by Ethyl Methane Sulfonate

**DOI:** 10.3389/fpls.2017.00017

**Published:** 2017-01-19

**Authors:** Xiao Xi-ou, Lin Wenqiu, Li Wei, Gao Xiaoming, Lv Lingling, Ma Feiyue, Liu Yuge

**Affiliations:** ^1^South Subtropical Crops Research Institute, Chinese Academy of Tropical Agricultural Sciences (CATAS)Zhanjiang, China; ^2^Zhanjiang City Key Laboratory for Tropical Crops Genetic ImprovementZhanjiang, China

**Keywords:** anthocyanin, chlorogenic acid, eggplant, EMS mutant, M_2_ generation, phenotypic variation

## Abstract

The eggplant was mutagenized with ethyl methane sulfonate (EMS) to enhance its genetic variability in our previous paper. In this article, we further analyzed the phenotype of M_2_ generation of mutant eggplants. A total of 325 independent M_2_ families were investigated for phenotypic variation. In addition to the visible phenotypic variation, chlorogenic acid (CGA) concentrations were analyzed in 26 fruits of mutants with High Performance Liquid Chromatography assay. Seventeen fruits exhibited significantly higher concentrations of CGAs than those in wild-type. The anthocyanin concentration of S9-1, the purple black mutant, was higher than WT, meanwhile, the anthocyanin concentration of L6-4 and U36-1 was lower than WT. Furthermore, our RT-PCR result demonstrated that the expression levels of anthocyanin biosynthetic genes, except for SmPAL, were increased in S9-1, and the regulator *SmMYB1* was decreased in L6-4 and U36-1 mutants. Together, our data indicated that, M_2_ generation showed abundant phenotypic variations and the strong potential usage for next step of breeding and molecular genetic mechanisms in eggplant.

## Introduction

Eggplant (*Solanum melongena* L.), which belongs to family Solanaceae, is a common vegetable in subtropic and tropic areas. The eggplant fruit contain abundant nutrient, such as phenolics compounds, protein, carbohydrates, mineral substance, and vitamin, which all were beneficial for human health. (San José et al., 2013). The eggplant phenolic compounds, such as anthocyanin and chlorogenic acid (CGA), have potential to scavenge reactive oxygen species. (Noda et al., 2000; Whitaker and Stommel, 2003; Hanson et al., 2006). The content of CGA in eggplant fruit were various between the eggplant cultivar and cultivated condition. The highest content of CGA in eggplant fruit was 28.0 g/kg dw as much as that in coffee (Mennella et al., 2012; Plazas et al., 2013). The delphinidin 3-rutinoside is the major form of anthocyanin found in the fruit peel which was contribution to the eggplant fruit color(Zhang et al., 2014). Nasunin which isolated from the eggplant fruit peels might be useful to prevent angiogenesis-related diseases (Matsubara et al., 2005). The eggplant has high fiber and low soluble carbohydrate content. Thus, an eggplant-based diet is recommended by the National Diabetes Education Program of NIH, Mayo Clinic, and American Diabetes Association for the management of type 2 diabetes and hypertension (Kwon et al., 2008). Otherwise, the eggplant fruit contain abundant of protein, vitamin C, mineral, dehydroascorbic acid Ayaz et al. (2015).

Although, the eggplant present diversity morphological, but the genetic of the cultivar eggplant is narrow (Meyer et al., 2012). Inducing mutations by chemical and physical methods is a highly efficient approach to increase genetic diversity (Shirasawa et al., 2016). Mutants are also potential materials for breeding new cultivar (Takagi et al., 2015). Moreover, mutants are also powerful tools for gene clone and function analysis by reverse or forward approach (Emmanuel and Levy, 2002; Takagi et al., 2013, 2015; Rizal et al., 2015).

Ethyl methane sulfonate (EMS) is one of the most popular chemical mutagens that induce mutations in plants, such as tomato (Saito et al., 2011; Shikata et al., 2016), *Arabidopsis* (Martin et al., 2009), and pepper (Hwang et al., 2014; Arisha et al., 2015). The EMS-induced mutants display improved traits, such as abiotic stress, phenotypic trait, and metabolite content. In rice, a salt-tolerant mutant was identified in 6,000 mutants (Takagi et al., 2015). A light-green exocarp mutant was discovered from the EMS-mutagenized cucumber line 406 with dark-green exocarp (Zhou et al., 2015). In soybean, the protein, oil, and sugar contents of the mutants are abundant (Tsuda et al., 2015). Although the EMS-induced mutation library shows abundant variations in the phenotypic trait and metabolic product content (Saito et al., 2011; Hwang et al., 2014; Arisha et al., 2015; Shikata et al., 2016), only the dominant mutation phenotypic traits are visualized in the M_1_ generation. In the M_1_ generation, the most identified characters are plant height, leaf color, and male sterility (Arisha et al., 2015). In the M_2_ generation, the recessive character is identified in the mutational base was composition homozygosis. Thus, the most efficient time to screen the mutant by forward or reversed methods should be in the M_2_ generation (McCallum et al., 2000; Takagi et al., 2013). Mutant phenotypes may not be inherited by the offspring because of DNA self-repair mechanism (Saito et al., 2011). Consequently, mutants in the M_3_ or M_4_ generations should be analyzed.

Next-generation sequencing is a powerful tool for analyzing the EMS-induced mutation (Gady et al., 2009; Uchida et al., 2011; Takagi et al., 2013; Henry et al., 2014). The types of EMS-induced mutation include SNV (base transition, base insertion, and base deletion), CNV, and indel section. The C/G to T/A transitions are the predominant mutations in EMS mutants (Uchida et al., 2011; Henry et al., 2014; Tsuda et al., 2015; Shirasawa et al., 2016). These mutations affect the protein synthesis or structure, thereby leading to phenotypic change. According to the base mutation effects on protein, the mutation could be divided into non-sense, frame shift, intron and intergenic, and synonymous mutations (Shirasawa et al., 2016). The whole genome sequencing result showed that intron and intergenic mutations are the predominant mutations (Shirasawa et al., 2016). Compared with the time- and labor-consuming map-based cloning methods, Mutmap technology, which is based on the mutants and whole genome sequencing, is an efficient and convenient approach in gene cloning (Takagi et al., 2013, 2015). In rice, several genes were cloned by the Mutmap methods such as *hst1*, (Takagi et al., 2015), *ppi* (Takagi et al., 2013). In cucumber, a new gene (gl2) conferring the Glabrous Trait in cucumber was identified using MutMap (Mengnan et al., 2015). The eggplant contains 24 chromosomes and the genome size was 1.13 GB. 85,446 genes were predicted in the genome which approximately 90% of the gene space was estimated (Hirakawa et al., 2014). The whole genome sequence of eggplant provides a power tool for eggplant breeding and the molecular mechanism researches.

In tomato, several mutant libraries in different backgrounds, such cultivars Micro-Tom, (Meissner et al., 1997; Saito et al., 2011), Red Setter (Gady et al., 2009), Tpaadasu (Minoia et al., 2010), and M82 (Menda et al., 2004), are available. Saito et al. (2011) created the Micro-Tom mutants and shared it (Saito et al., 2011; Shikata et al., 2016). Compared with other species of Solanaceae family, such as tomato and pepper, the mutant libraries research on eggplant is relatively limited. In our previous study, a highly homozygous inbred line E31-1 was treated by 1.0% EMS (V/V), and the physiological variations in M_1_ generation were analyzed (Xiao et al., 2016). The E31-1 inbred line fruit length is about 35 cm and the color is purple. In this article, we further investigate the M_2_ generation phenotypes in this EMS mutagenized line, and analyzed the CGA and anthocyanin concentrations. Our works may have considerable significance in eggplant breeding and molecular mechanisms research.

## Materials and Methods

### Plant Material

In our previous study, we used 1% EMS (V/V)-mutagenized eggplant seeds (20 g), and 790 families of M_2_ generation seeds were harvested (Xiao et al., 2016). 325 of 790 families were analyzed in this study. For each independent line, four individual plants were transplanted, but more than 150 M_2_ plants die. 1142 M_2_ plants were transplanted in the field located in the South Subtropical Crop Research Institute Chinese Academy of Tropical Agricultural Sciences (21°10′2″ N; 110°16′34″ E). The M_2_ generation phenotypic traits were investigated and recorded.

### CGA Measurement with HPLC Assay

The CGA content in fruit was analyzed by HPLC when the fruit was commercially ripe. Fresh samples (1 g) were homogenized in 5 ml of 80% methanol. Subsequently, the extract was sonicated for 1 h at room temperature and centrifuged at 2000 rpm for 3 min. The supernatant was filtered through 0.45 μl nylon membrane filters. The CGA content was detected by HPLC (LC-20A Shimadzu Japan). Approximately 10 μl of extracts were injected using LC system automatic sampler into an Eclipse XDB-18 (5 μl, 4.6 mm × 25 mm; Agilent Technologies) column protected by an Eclipse XDB-C18 (5 μl, 4.6 mm × 12.5 mm grd car 4/PK; Agilent Technologies). The method was performed according to that of Plazas et al. (2014). The binary gradient consisted of A (0.1% formic acid, HPLC-grade; Sigma) and B solutions (100% methanol, HPLC grade; Sigma). The following conditions were observed: 0 min, 95 A:5 B; 0–3 min linear increase to 10% B; 3–6 min, linear increase to 20% B; 6–12 min, linear increases to 83% B; 12–16 min, linear increase to 100% B; 16–20 min, 100% B; 20–21 min, decrease to 5% B; and 31–31 min, 95 A:5 B. The B flow was 0.8 ml/min. Quantification was based on absorbance at 325 nm.

### The Measurement of Anthocyanin

The total anthocyanin of fruit peel was detected by UV-Visible Spectroscopy according to Giusti and Wrolstad (2001) and Zhang et al. (2014). About 100 mg of pericarps were powdered in liquid nitrogen and extracted in 2 ml of 1% HCl in methanol. After centrifugation at 14000 *g* for 10 min at 4°C, 0.2 ml of supernatant was added to 2 ml solutions A (25 mM KCl, pH 1.0) and B (0.4 M sodium acetate buffer, pH 4.5). The absorbances at 543 and 700 nm were measured. The total anthocyanin content was calculated as follows: anthocyanin pigment (mg/g.FW) = [(A_543_ - A_700_)_pH 1:0_ - (A_543_ - A_700_)_pH 4:5_ × 465 × 10]/ (29000×1). The molecular mass and molar absorptivity of Dpd-3-glu at 543 nm was 465 and 29000, respectively.

### RNA Isolation and Quantitative PCR

The total RNA of the fruit peel was extracted using the column plant RNAout2.0 kit manual (Tian Enze Beijing). Approximately 1 μg of RNA was synthesized into cDNA with Oligo dT_18_, according to the manufacturer’s instruction (Takara Dalian). The gene expression was analyzed by using Roche LightCycler 480 thermal cycler. About 10 μl of reaction mix contained 5 μl of 2X Maxima SYBR Green qPCR Master Mix (Thermo fisher), 2 μl of primers, 1 μl of cDNA, and 2 μl of RNase-free water. The amplification program was as follows: 95°C for 3 min, 95°C for 15 s, 60°C for 30 s, and 72°C for 15 s, 45 cycles. The primers used in this study were listed in Supplementary Table S1 and the method was in accordance with Zhang et al. (2014).

## Result

### M_2_ Plant Phenotyping

**FIGURE 1:**
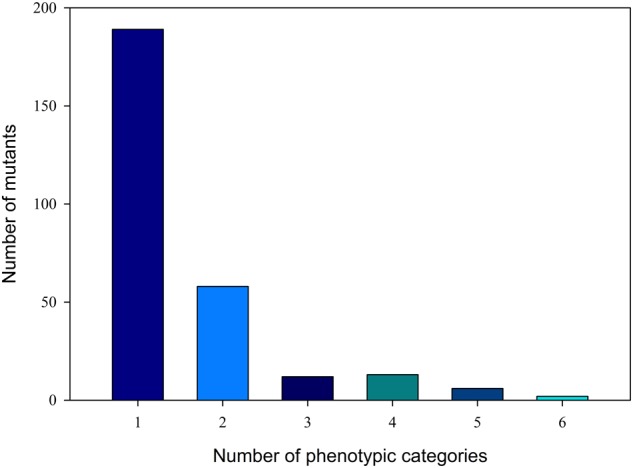
**Distribution of phenotypic categories of M_2_ generation.** The *x*-axis shows the phenotypic categories number, and the *y*-axis shows the number of mutants in the relevant category.

M_2_ plant phenotyping was inspected in visible phenotypes. All visible phenotypes were divided into 15 major categories and 38 secondary categories (**Table 1**). A total of 443 phenotypic categories were investigated. According to the visible phenotypes, a total of 280 mutants were identified from the 1142 M_2_ plants. **Figure 1** shows that 189 of the 280 mutants presented only one phenotypic category, and 91 mutants showed more than one phenotypic category. Most mutants, such as the L6-4 mutant, contained six phenotypic categories, namely, high height value, green stem, green vein color, green fruit peel, violet flower color (Supplementary Figure S1), and green fruit flesh color (Supplementary Figure S2).

**Table 1:** List of phenotypic categories and the number of phenotypes.

First category	Secondary category	No.
Plant height	1 Dwarf (30 cm)	20
	2 Short (30–60 cm)	53
	3 Tall (>120 cm)	4
Plant habit	1 Internode length	3
	2 Branching	23
	3 Other plant habits	4
Stem color	1 Purple	4
	2 Green	7
Leaf morphology	1 Leaf size	13
	2 Leaf texture	23
	3 Leaf margin	1
	4 Leaf shape	8
	5 Vein color	7
	6 Other leaf morphologies	19
Leaf color	1 Yellow-green	4
	2 Dark-green	6
	3 Variegation	2
	4 Pale green	8
Flowering timing	1 Early	6
	2 Late	52
Flower color	1 Violet	3
Flower size	1 Large	4
	2 Small	0
Sterility	1 Full sterility	Nr
Fruit size	1 Small	8
	2 Long	10
	3 Short	41
	4 Large	12
Fruit shape	1 Oval	2
	2 Other shapes	14
Fruit flesh color	1 Green	5
Fruit number	1 Many (Fruit number >3)	18
Fruit color	1 Green	3
	2 Purple-black	42
	3 White	4
	4 Other colors	1
Calyx color	1 Green	9
Total		443

**FIGURE 2:**
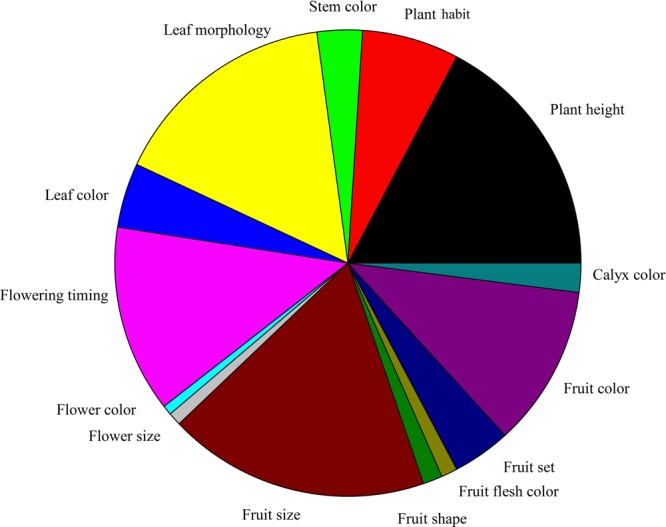
**Classification of visible mutant phenotypes.** The 15 major categories and the number of phenotypic categories in the M_2_ generation of eggplant EMS-mutagenized population.

**Figure 2** illustrates the classification of visible mutant phenotypes by 15 major phenotypic categories. The most abundant phenotypic category was fruit size (18.53%), which was followed by plant height (17.62%). The category with the fewest phenotypes was flower color (0.68%).

### Chlorophyll Mutations in M_2_ Generation Seedling

**FIGURE 3:**
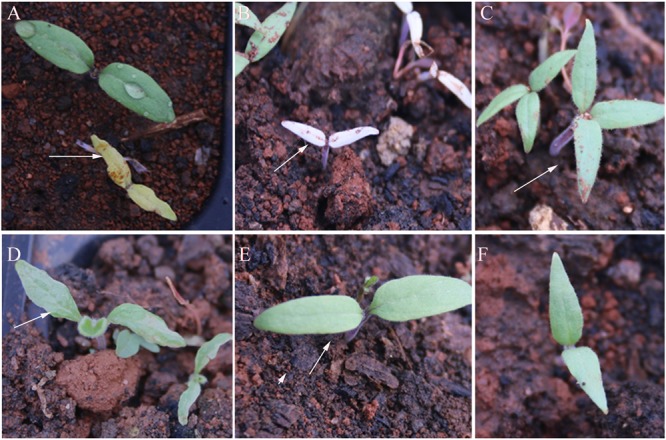
**Mutant characteristics of cotyledons of M_2_ generation. (A)** Yellow seedlings (totally yellow), **(B)** Albino seedlings (totally white), **(C)** three-cotyledon, **(D)** cotyledons malformation, **(E)** large cotyledons, and **(F)** wild-type (WT). The arrows indicated the mutant.

Chlorophyll and cotyledon mutations were the first visible mutant characters. **Figure 3** shows the representative characteristics of mutant seedlings, including yellow color (seven families), albino (three families), three cotyledons (one family), cotyledon malformation (two families), and large cotyledons (four families).

### Plant Height

**FIGURE 4:**
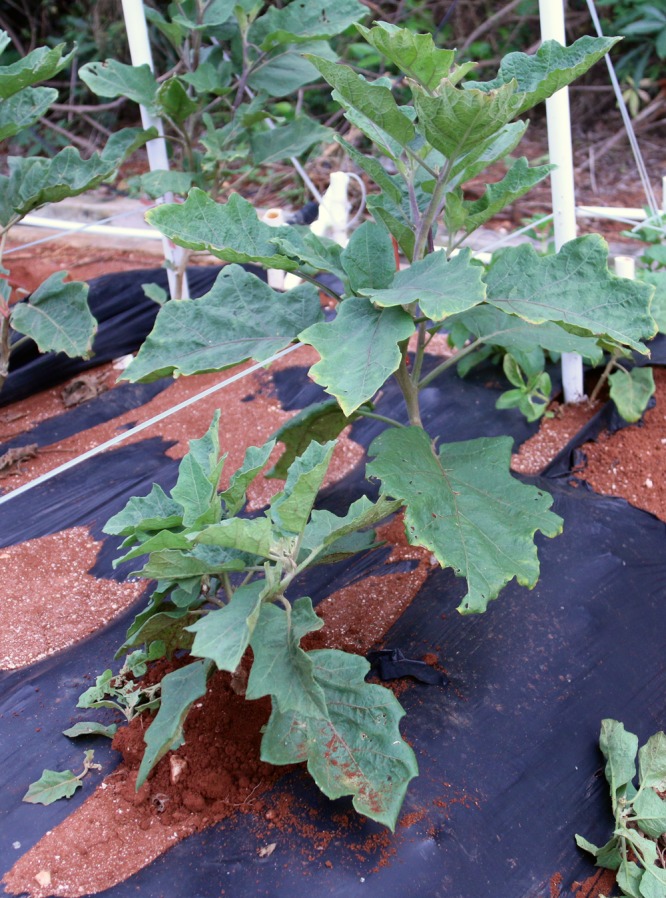
**Dwarf plant compared with WT plants during M_2_ generation.** The dwarf mutant was K50-3 which height was 25 cm. The photo was taken at 90 days after transplanting.

The plant height of mutants was divided into dwarf, short, and tall categories. The smallest mutant was K50-3 at 25 cm with short internodes (**Figure 4**). K50-3 mutant also showed small leaves and fruit size. The tallest mutant was L6-2 (123.4 cm), which also showed longer internodes and larger leaves compared with the wild-type (WT) eggplant.

**FIGURE 5:**
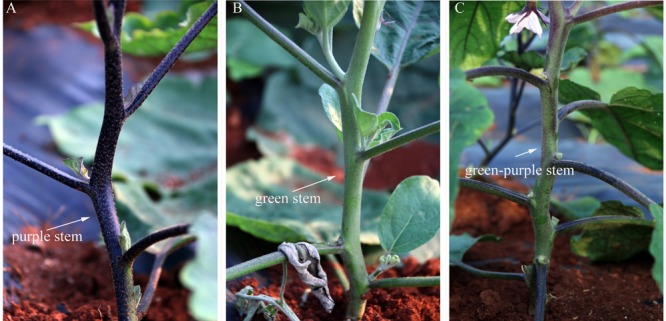
**Stem color change in the M_2_ generation. (A)** W29-1 developed purple stem, **(B)** A18-6 developed green stem, **(C)** WT presented green-purple stem.

**Figure 5** shows two categories of stem color in the mutant. WT eggplant showed green-purple stem, whereas W29-1 and A18-6 mutants showed purple and green stems, respectively (**Figure 5**). Moreover, W29-1 showed less hair than those of A18-6 and WT eggplant. The purple and green-stemmed mutants also showed purple and green petiole and leaf vein.

### Leaf Structure

**FIGURE 6:**
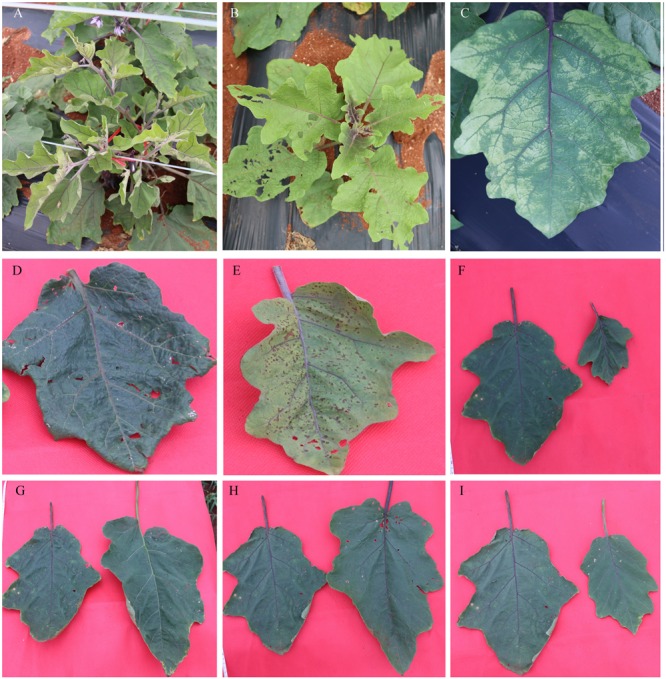
**Representative mutants relate to leaf color and morphology. (A)** U41-3 developed yellow-green leaves, **(B)** 25-5 developed yellow-green leaves, **(C)** O1-2 mutant developed yellow-spotted leaves, **(D)** W42-8 developed dark-green and lesion-like pointed leaves, **(E)** R34-1 developed yellow-green and disease spot-like leaves. **(F)** W38-1 developed abaxially curled, small leaves, **(G)** L6-1 developed large, green leaf with green vein, **(H)** N36-1 developed long and narrow leaves, **(I)** 55-7 developed shallow leaf margin and small leaves. The leaf on the left in **(F–I)** represents the WT leaf.

**FIGURE 7:**
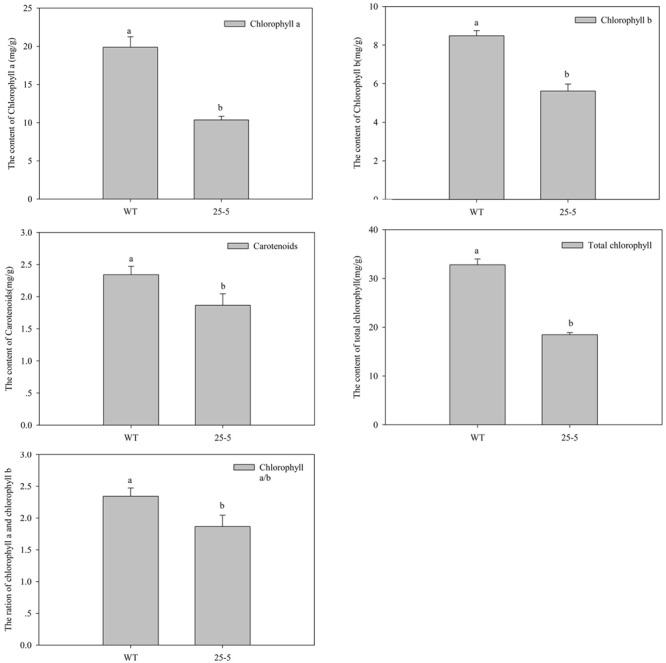
**Analysis of Chlorophyll content between WT and 25-5 mutant plants.** The 25-5 mutant developed yellow green leaves. Different letters indicate significance at *P* < 0.05.

**Figure 6** shows the representative mutants of leaf color and morphology. Compared with the WT eggplant, the leaf color, size, and morphology showed abundant mutation. **Figure 6A** (U41-3) and **Figure 6B** (25-5) show different degrees of yellow leaf mutation. The O1-2 mutant developed yellow-spotted leaves (**Figure 6C**). W42-8 developed dark-green and lesion-like pointed leaves (**Figure 6D**). Furthermore, R34-1 mutant developed yellow-green and disease spot-like leaf (**Figure 6E**), and W38-1 developed abaxially curled, small leaves (**Figure 6F**). L6-1 developed large, green-leaf vein leaf (**Figure 6G**). N36-1 developed long and narrow leaf (**Figure 6H**). 55-7 developed shallow leaf margin and small leaves (**Figure 6I**). **Figure 7** illustrates that chlorophyll a, chlorophyll b, total chlorophyll, and ratio of chlorophyll a/b in leaves of the WT eggplant were significantly higher than in leaves of the yellow leaf mutant 25-5. However, the carotenoid content in WT eggplant and 25-5 leaves was similar.

### Fruit Mutants

**FIGURE 8:**
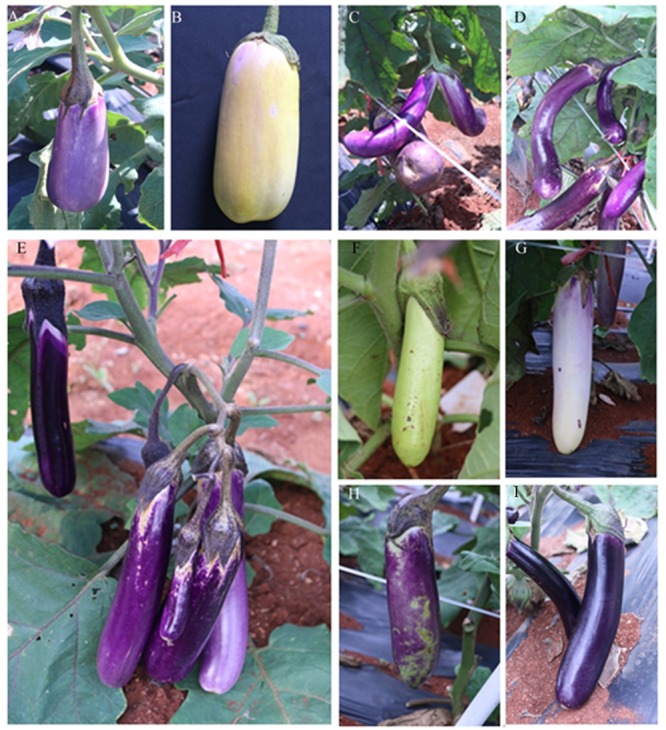
**Representative mutants relate to fruit color and morphology. (A)** V40-1 developed a small and short fruit, **(B)** 48-5 developed large eggplant fruit, **(C)** 55-3 developed malformed fruit, **(D)** A3-6 developed curve eggplant fruit, **(E)** P47-3 bore many fruits, **(F)** L6-4 developed green eggplant fruit peel, **(G)** U36-1 developed a white eggplant fruit peel, **(H)** L6-2 developed variegated fruit peel color, **(I)** 55-6 developed purple-black fruit peel.

**Figure 8** presents the representative fruit mutants in M_2_ generation. A total of 81 phenotypic categories were observed for fruit size, such as small and short (**Figure 8A**), large fruit (**Figure 8B**), malformed (**Figure 8C**), and curve eggplant fruits (**Figure 8D**). A total of 18 mutants showed many fruits at the first node (**Figure 8E**). Additionally, the mutant showed various fruit peel colors, such as green, white, variegated, and purple black (**Figures 8F–I**).

### CGA Content Variation in M_2_ Mutants

**FIGURE 9:**
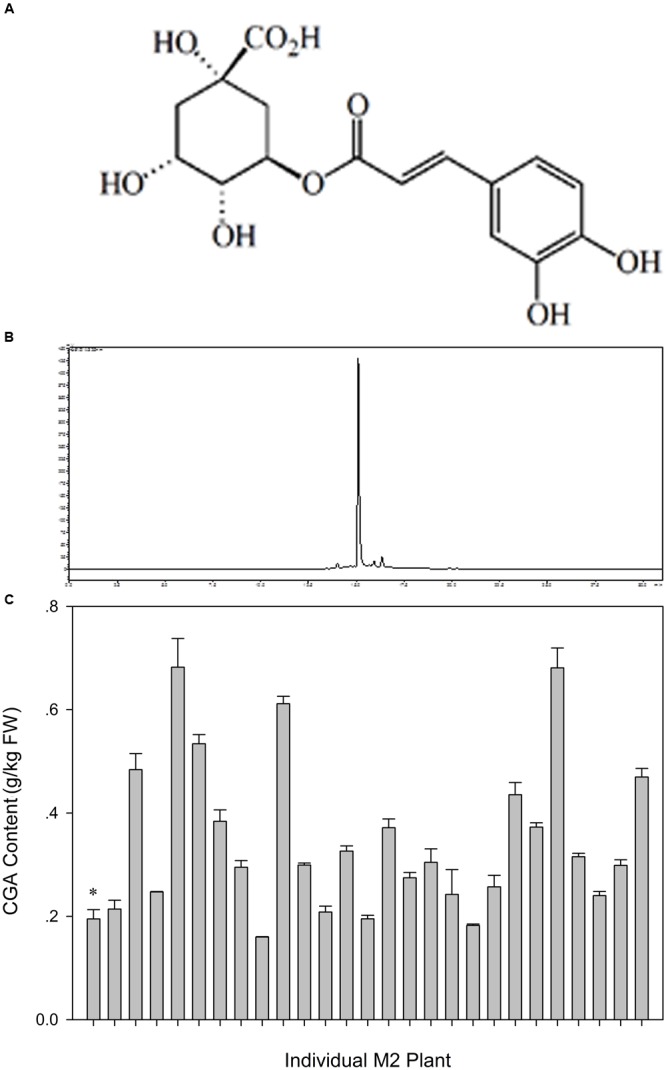
**Variation in CGA content among fruits in M_2_ generation. (A)** Chemical structure of CGA, **(B)** CGA content analyzed by HLPC method, **(C)** Variation in CGA content among fruits of M_2_ plant. The *x*-axis shows the mutant, and the *y*-axis shows the content of CGA. The bar showed the content of CGA in the mutant. ^∗^ indicates the WT eggplant.

The CGA content in M_2_ fruit is presented in **Figure 9**. The CGA content in the library significantly shifted to higher values (**Figure 8**) compared with WT plants (0.1950 ± 0.0180). The CGA contents of 17 out of 26 M_2_ plants were significantly higher than in WT eggplant fruit. The maximum CGA content was 0.6825 ± 0.0552, which was 3.5-fold higher than that of WT eggplant fruit.

### Variation in Anthocyanin Content in Fruit Peel of Mutants

**FIGURE 10:**
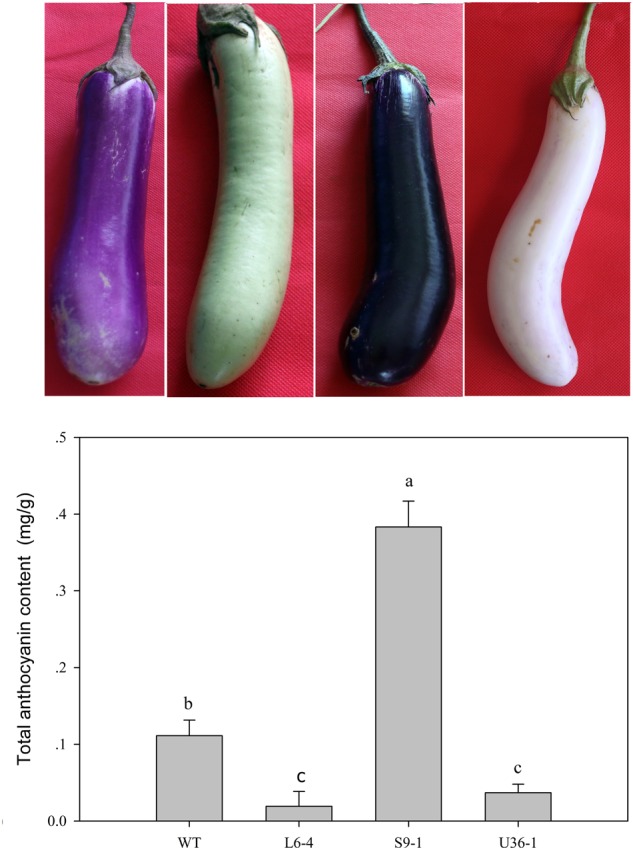
**Total anthocyanin content change in the M_2_ generation.** WT fruit showed the purple color, the S9-1 mutant showed purple black color, the L6-4 mutant showed green fruit color, the U36-1 mutant showed the white color. Statistical significance of the differences between samples was calculated with ANOVA by paired-group comparisons. Different letters indicate significance at *P* < 0.05.

The anthocyanin contents of WT, purple-black (S9-1), green (L6-4), and white (U36-1) eggplants were detected. The anthocyanin content of purple-black (S9-1) was higher than that in WT eggplant (**Figure 10**). The minimum level of anthocyanin content was observed in green eggplant (L6-4), which was 17.27% of that in wild type (**Figure 10**).

### Expression of Anthocyanin Biosynthetic and Regulatory Genes

**FIGURE 11:**
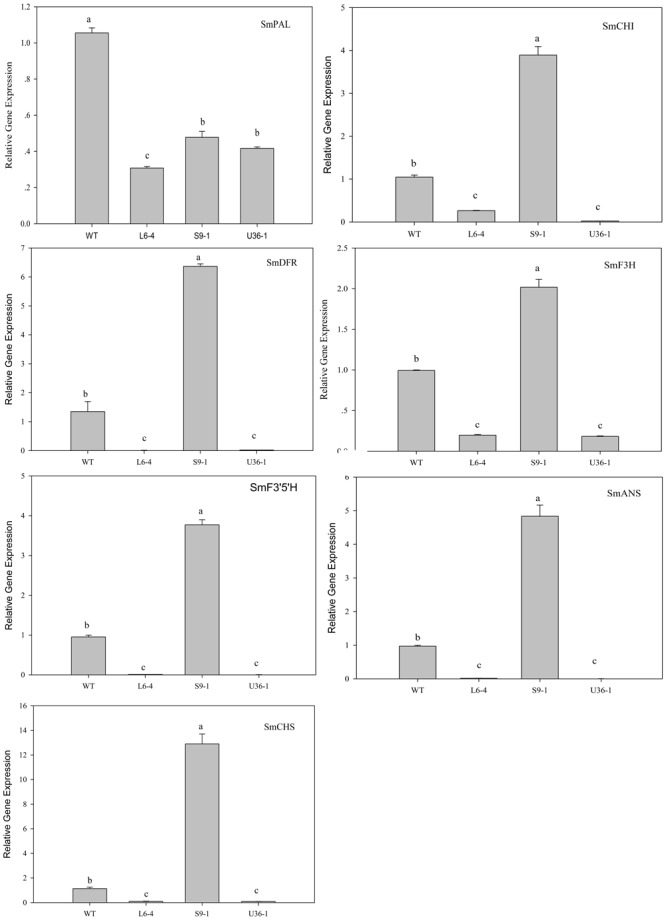
**Expression of anthocyanin biosynthetic genes in fruit peel of the WT and mutant eggplant in the M_2_ generation.** WT fruit showed the purple color, the S9-1 mutant showed purple black color, the L6-4 mutant showed green fruit color, the U36-1 mutant showed the white color. Different letters indicate significance at *P* < 0.05.

**FIGURE 12:**
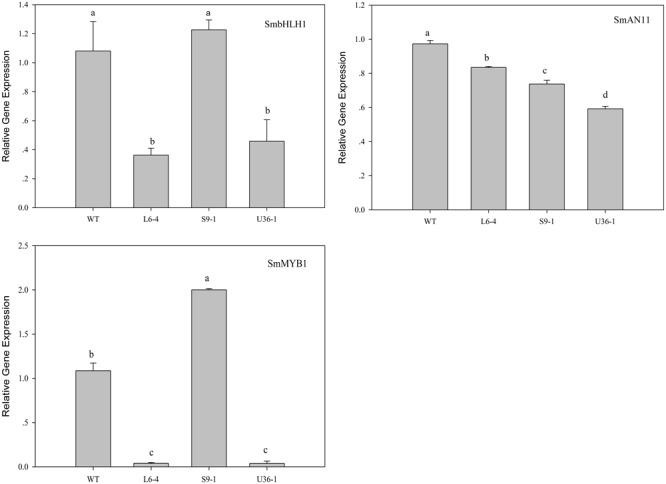
**Expression of anthocyanin regulatory genes in fruit peel of the WT and mutant eggplant in the M2 generation.** WT fruit showed the purple color, the S9-1 mutant showed purple black color, the L6-4 mutant showed green fruit color, the U36-1 mutant showed the white color. Different letters indicate significance at *P* < 0.05.

The expression of anthocyanin biosynthetic and regulatory genes in fruit peel was analyzed by RT-PCR. **Figure 11** shows that the expression level of anthocyanin biosynthetic genes *SmCHI, SmDFR, SmF3H, SmF3′5′H, SmANS*, and *SmCHS*, but not *SmPAL*, was significantly increased in S9-1 compared with the WT. Furthermore, the expression levels of *SmPAL SmCHI, SmDFR, SmF3H, SmF3′5′H, SmANS*, and *SmCHS* in L6-4 and U36-1 were decreased significantly compared with the WT one. The expression level of anthocyanin regulatory gene *SmbHLH, SmMYB1*, and *SmAN11* was also analyzed by using RT-PCR. **Figure 12** shows that the expression level of *SmbHLH* in WT and S9-1 was higher than that in L6-4 and U32-1. The maximal expression level of *SmAN11* was in WT eggplant fruit root, and followed by that in L6-4, S9-1, and U32-1. Nevertheless, the *SmMYB1* expression in S9-1 was significantly increased and decreased in L6-4 and U32-1 compared with WT.

## Discussion

Ethyl methane sulfonate-induced mutation is a powerful tool for innovation germplasm resources. Despite the many reports about EMS-induced mutation in plant, few researches have been conducted on the EMS-induced mutation in eggplant. To innovate the germplasm resources, eggplant seed was treated with 1.0% EMS previous (Xiao et al., 2016), and the M_2_ generation was analyzed in the present study. The mutants of M_2_ generation showed abundant visible mutant phenotypes, such as plant height, leaf color, and fruit peel color. They also showed various content metabolites, such as CGA and anthocyanin.

In the M_2_ generation, the recessive character should be present such as the yellow and albino seedling. M_2_ generation showed abundant mutant phenotypes and the mutation frequency of the phenotypes were different. The plant height phenotype showed the highest mutation frequency, whereas the flower phenotype showed the lowest mutation frequency. A similar phenomenon was reported by Saito et al. (2011) and Tsuda et al. (2015). This phenomenon may be explained by the fact that the higher mutation frequency phenotypes were regulated by the more number of structure or regulatory genes. In the last decades years, the fruit size, color and shape were the mainly goals for the eggplant breeders (Kashyap et al., 2003). In the present study, some of the mutants were potential benefit for eggplant breeding, such as fruit size mutant (48-5), fruit color mutant (S9-1). Furthermore, the fruit size and color mutants are crucial to understanding the regulatory mechanisms for fruit size and color development.

Delphinidin is the major anthocyanin type in eggplant fruit peel, and it contributes to the fruit peel color (Noda et al., 2000; Matsubara et al., 2005; Zhang et al., 2014). In this study, the M_2_ generation presents four types of fruit peel color, and the pH differential methods result showed that the order of anthocyanin content in the mutants was as follows: S9-1 > WT > U32-1 > L6-4. To further analyze the molecular mechanism underlying the change of fruit peel color, the known anthocyanin biosynthetic and regulatory gene (Zhang et al., 2014) expression level was detected by RT-PCR. The expression level of six biosynthesis gene, but not SmPAL, increased in S9-1 and decreased in L6-4 and U32-1 compared with WT. Zhang et al. (2014) also showed that the biosynthetic gene expression, except for SmPAL, was unregulated in the fruit peel of the purple eggplant cultivar. Those result suggested that SmPAL may not be involved in the eggplant anthocyanin biosynthetic pathway. Compared with the WT eggplant which fruit peel was black color, the expression of *CHS, DFR*, and *ANS* in a spontaneous green color mutant were significantly lower (Gisbert et al., 2016).

Several results indicated that *MYB* and *bHLH* transcriptional factor families play a vital role in the regulation of tissue color (Umemura et al., 2013; Zhu et al., 2015). Gisbert et al. (2016) showed that the regulatory genes (*MybC, Myc, and Wd*) was significantly decreased in the green color mutant in comparison with the WT eggplant. MYB1 positive regulates the anthocyanin accumulation in eggplant which was proved by overexpression experiment and the *MYB1* upregulate most anthocyanin biosynthetic genes (Zhang et al., 2014). Meanwhile the whole genome sequence analyze result indicated two MYB-like gene may involved in the eggplant anthocyanin synthesis (Hirakawa et al., 2014). In the present study, only the SmMYB1 gene expression level increased in S9-1 and decreased in L6-4 and U32-1. To further analysis the mechanism of the different color present in the mutants, the *SmMYB1* and *SmbHLH* gene were cloned and analyzed. The result showed that there is no mutation of *SmMYB1* and *SmbHLH* between the WT and S9-1, L6-4 and U32-1 (Supplementary Figures S3 and S4). The result suggested that the mutational genes regulate the MYB1 and most of anthocyanin biosynthetic genes to regulate the anthocyanin accumulation. The genetic experiment result indicated that the fruit pigmentation was controlled by one or multiple genetic factors based on the material (Hirakawa et al., 2014; Gisbert et al., 2016). In our study, the L6 mutant present variegated fruit color. And the progeny of L6 line showed a different degree green color (Supplementary Figure S5). The segregation law was not compatible with Mendel inheritance models suggesting that the mutation responsible for the green color influences several genetic factors that control fruit anthocyanin accumulation.

Chlorogenic acid is beneficial to human health. The CGA content in eggplant is lower than that in coffee, and eggplant could be the CGA source in diet (Plazas et al., 2013). Compared with the results of a previous report (Plazas et al., 2014), the CGA content in the present study was relatively lower. However, the CGA content of the analyzed individual M_2_ plant was higher than in WT eggplant fruit. The key genes which involved in the CGA biosynthesis was revealed (Plazas et al., 2014). The homologous gene of the biosynthetic pathway is predicated in the eggplant genome (Gramazio et al., 2014; Hirakawa et al., 2014). Nevertheless, no experiment has been conducted to confirm the genes that regulate CGA biosynthesis. The M_2_ plant is not ideal breeding materials for breeding high content CGA eggplant variety, but it is a suitable material for analyzing the CGA biosynthetic mechanism in eggplant.

Despite that M_2_ generation showed abundant mutations, the mutant family number was still insufficiently. Emmanuel and Levy (2002) reported that approximately 130,000 plants of fast-neutron irradiation and a range of 10^3^ individuals are needed for a near-saturated mutant population of tomato for reverse genetics. In future studies, the M_2_ generation family should be expanded to obtain a near-saturated mutant population, and the genetic control mechanisms for the visible phenotypic changes should be analyzed.

## Author Contributions

XX-o designed the experiment and composed the manuscript. LWq analyzed the gene expression and revised the manuscript. LW and GX investigated and analyzed the phenotypic categories. LL analyzed the chlorophyll content and revised the manuscript. MF analyzed the CGA content. LY analyzed the anthocyanin content.

## Conflict of Interest Statement

The authors declare that the research was conducted in the absence of any commercial or financial relationships that could be construed as a potential conflict of interest.
