# Supplementary material for: The Analysis of Physiological Variations in M2 Generation of Solanum melongena L. Mutagenized by Ethyl Methane Sulfonate
Source: Front Plant Sci. 2017 Jan 19;8:17. doi: 10.3389/fpls.2017.00017 (PMC5243811; doi:10.3389/fpls.2017.00017)
Supplement: Supplementary file 1 [file Data_Sheet_1.docx]

Supplementary Material

The physiological variations in M_2_ generation of *Solanum melongena* L. mutagenized by Ethyl methane sulfonate

**Xiao Xi-ou*1,2, Lin Wenqiu1,2, Li Wei1,2 , Gao Xiaomin1,2, Lv Lingling1,2 , Ma Feiyue1,2, Liu Yuge1,2**

**,* Correspondence:**Xiao Xi-ou: xiao-forlearning@163.com

# Supplementary Figures and Tables

## Supplementary Figures


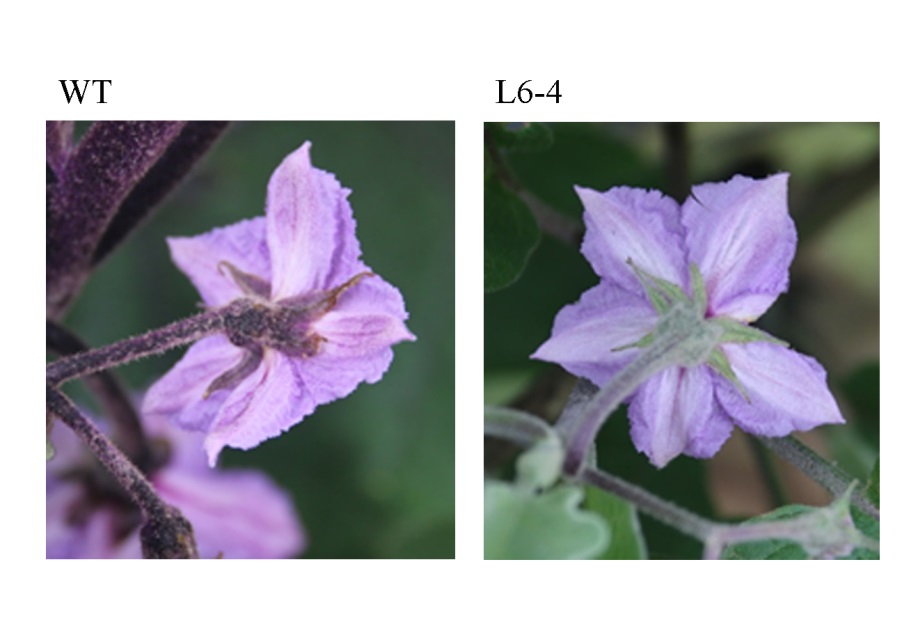


# Figure S1 The L6-4 mutant showed violet flower color


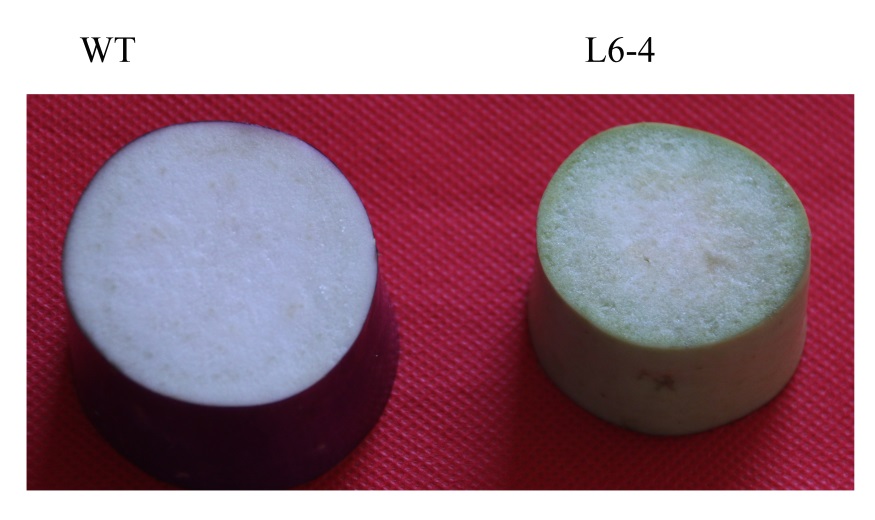


# FigureS2 The L6-4 mutant showed green fruit flesh color.

#
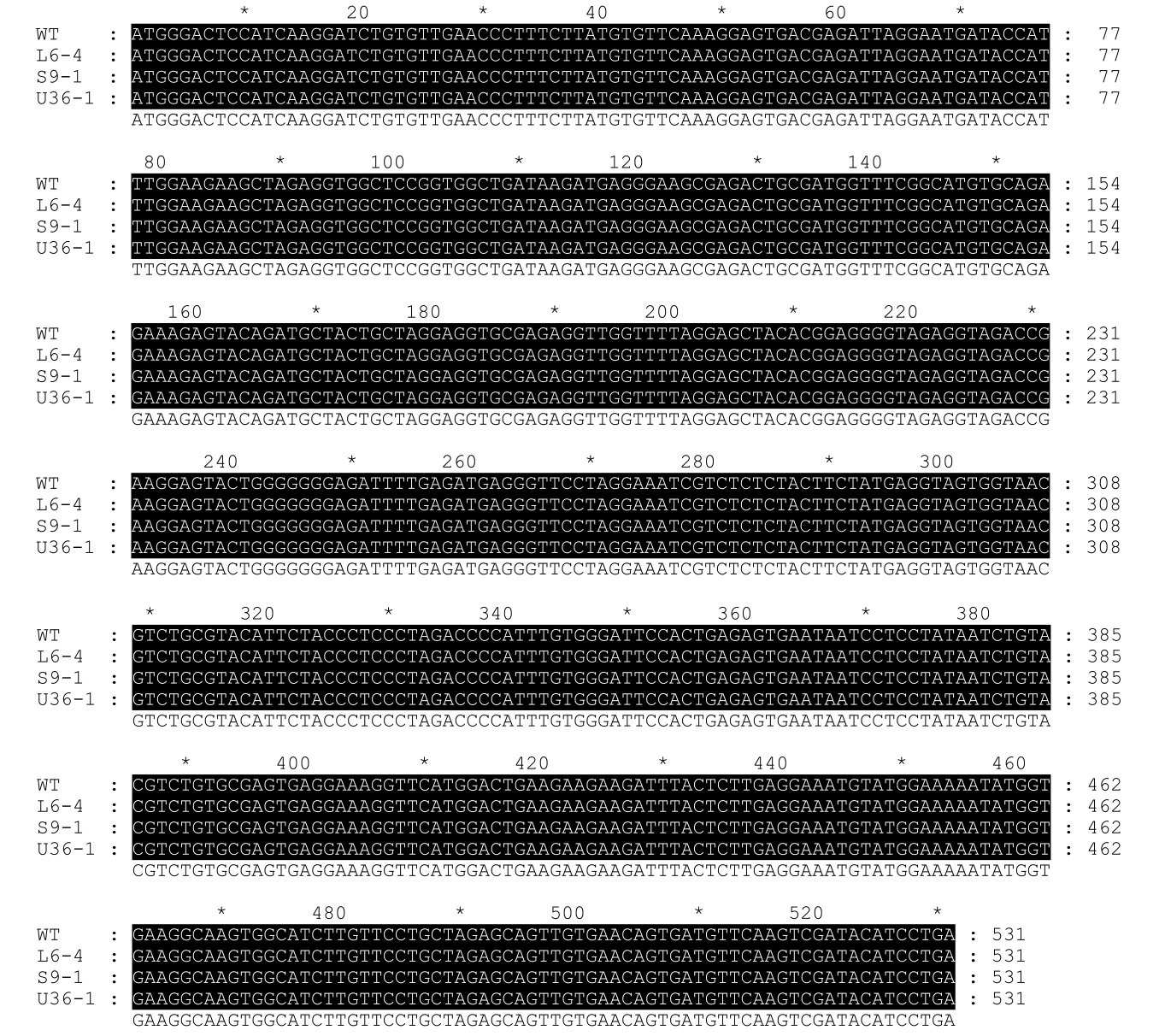


**Figure S3 The *SmMYB1* sequence alignment between WT L6-4,S9-1 and U36-1.** The S9-1 mutant showed purple black color, the L6-4 mutant showed green fruit color, the U36-1 mutant showed the white color.

#

#
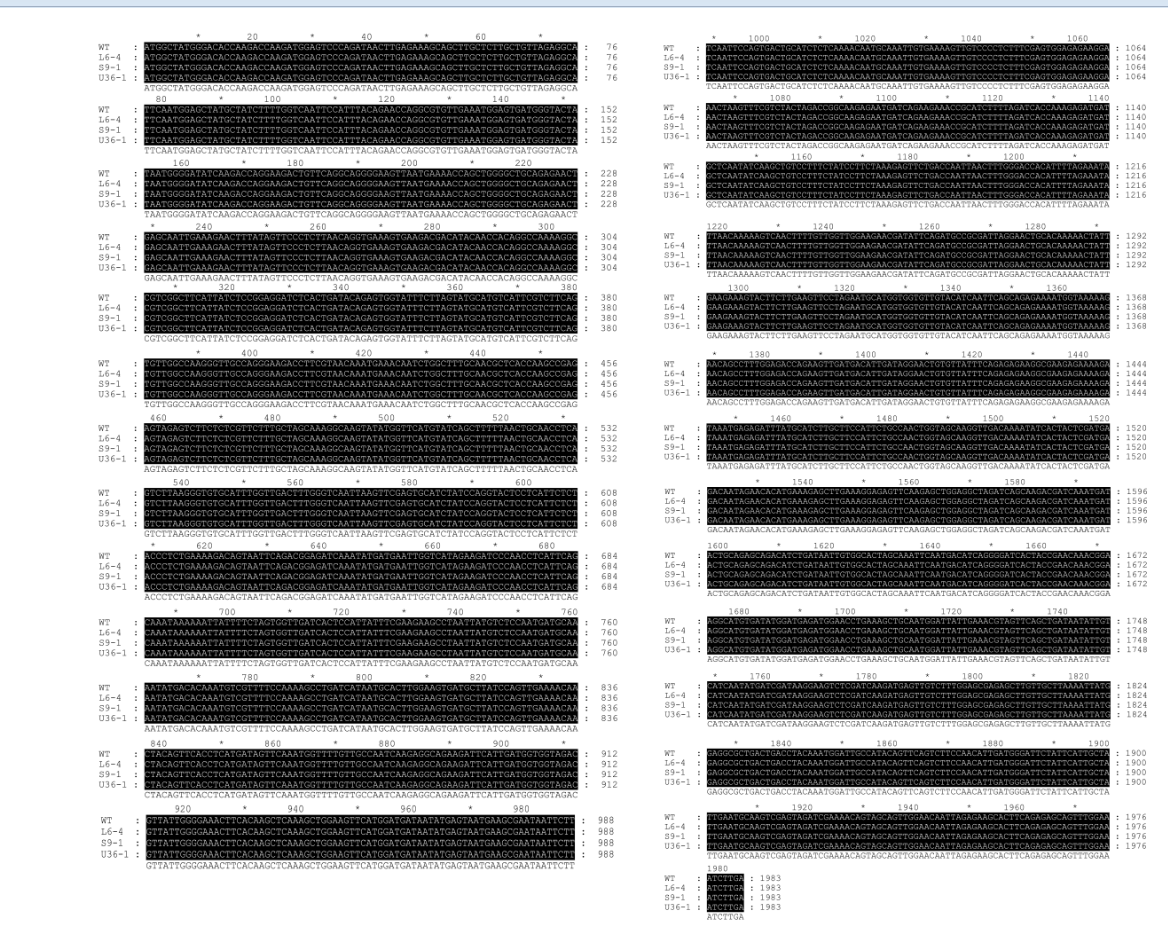
 Figure S4 The SmbHLH sequence alignment between WT L6-4,S9-1 and U36-1. The S9-1 mutant showed purple black color, the L6-4 mutant showed green fruit color, the U36-1 mutant showed the white color.

#
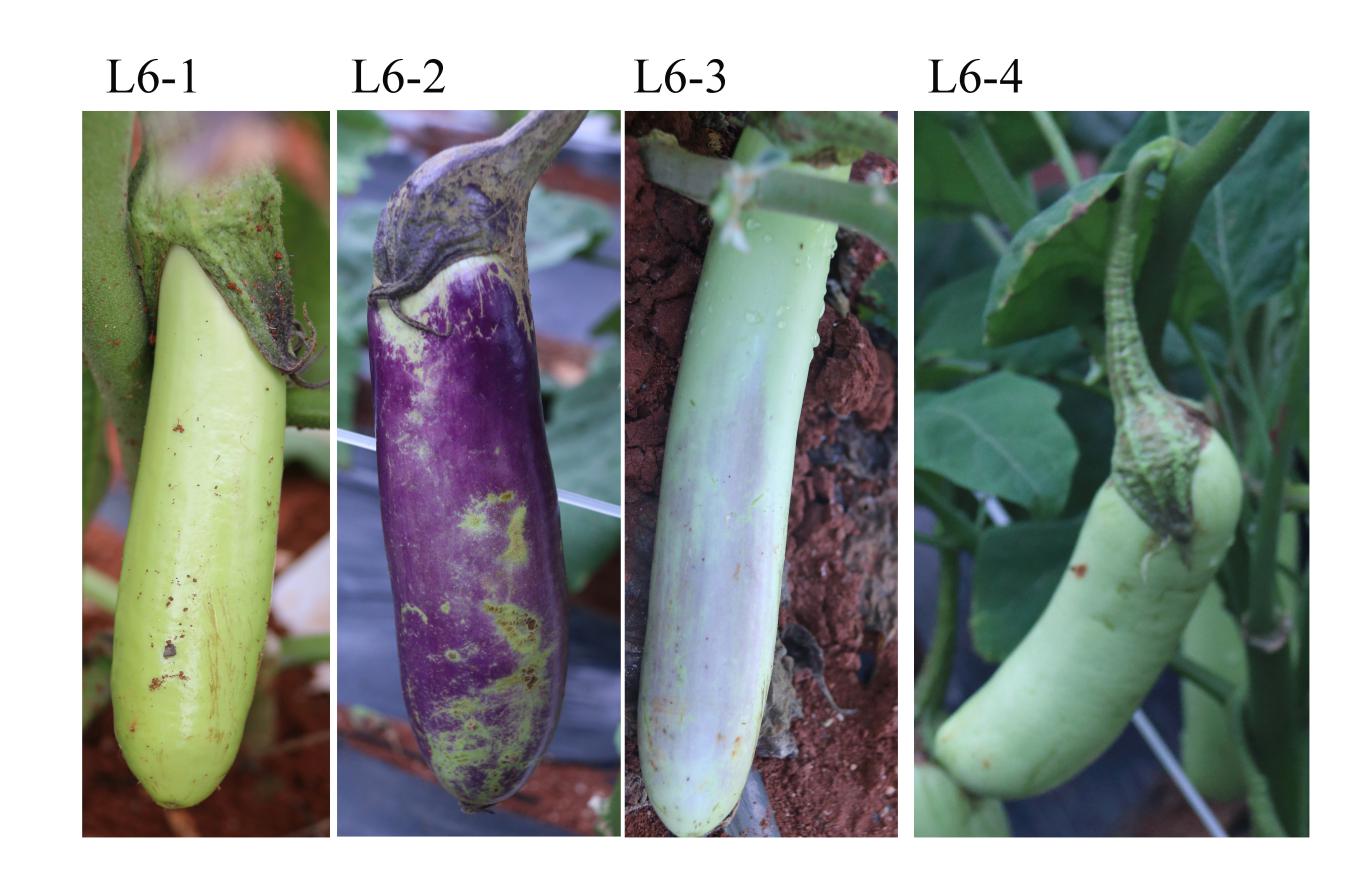


# FigureS5 The fruit peel color of the M_2_ generation of L6 line. The L6-1,L6-2,L6-3,L6-4 eggplant fruit showed different degree green color.

# Table S1 the primers used for RT-PCR

| Genes | Primers Sequence (5’-3’) |
| --- | --- |
| *SmPALF* | *TGCCTATGCTGATGATCCCT* |
| *SmPALR* | *GTTCCCACTTTCCAACGCTA* |
| *SmCHIF* | *AAATACAAAAGCCAGCATAACC* |
| *SmCHIR* | *AGGGGAGTGTCACCTACAACTA* |
| *SmF3HF* | *AATGCGATAGTGTATCCGTTAA* |
| *SmF3HR* | *CAAGCAAGAATTTCCTCAATG* |
| *SmF3'5'HF* | *CTCATCTAGCGTGATAGAATGG* |
| *SmF3'5'HR* | *TTTAGTGGCGTTGAAGGGT* |
| *SmDFRF* | *TTCATTTGCTCATCCCATC* |
| *SmDFRR* | *GCCCCTTGATACATATCCTC* |
| *SmANSF* | *GCACTGACTTTCATCCTCCAC* |
| *SmANSR* | *TCTTGTACTTTCCGTTGCTTAG* |
| *SmAN11F* | *TCGGTATCTGCTGATGGGTC* |
| *SmAN11R* | *TGTATCTCAAATCTTGCTTGTTCC* |
| *SmbHLHF* | *CTTTGGAGACCAGAAGTTGATG* |
| *SmbHLHR* | *CTTGCTACCAGTTGGCAGAAT* |
| *SmMYB1F* | *CAAGTGACAAGCAAACTACCG* |
| *SmMYB1R* | *TCTTCTCCTTCAACAGCGTC* |

# The primers referenced as Zhang et al.,
